# Pregnancy Outcomes After Different Cycle Regimens for Frozen-Thawed Embryo Transfer: A Retrospective Study Using Propensity Score Matching

**DOI:** 10.3389/fmed.2020.00327

**Published:** 2020-07-28

**Authors:** Bian Wang, Qianqian Zhu, Yun Wang

**Affiliations:** Department of Assisted Reproduction, Shanghai Ninth People's Hospital Affiliated to JiaoTong University School of Medicine, Shanghai, China

**Keywords:** live birth rate, endometrium preparing, modified natural cycle, artificial cycle, stimulated cycle, frozen–thawed embryo transfer

## Abstract

**Background:** Nowadays, the choice of frozen embryo transfer (FET) regimens is mainly guided by personal convenience. Clinicians prefer the predictability and reliability of artificial cycle (AC) FET and have extended its usage to general *in vitro* fertilization population. More recent primary studies are beginning to challenge the comparability of AC-FET and suggest reduced clinical pregnancy rate and live birth rate (LBR) compared with those in modified natural cycle (mNC) FET (ovulation triggered by human chorionic gonadotrophin) and stimulated cycle (SC) FET.

**Objective:** To assess the pregnancy outcomes within matched mNC-FET, SC-FET, and AC-FET cycles by using propensity score matching (PSM) in a larger cohort.

**Methods:** A total of 16,946 women who underwent their first autologous FET cycle between July 2014 and July 2017 were evaluated. PSM, using the nearest neighbor matching, were established to adjust the baseline features within the three protocols in proportion of 1:1 (mNC-FET vs. SC-FET, mNC-FET vs. AC-FET, SC-FET vs. AC-FET). Furthermore, there were 3,567, 2,917, and 3,964 cycles compared between matched mNC-FET and SC-FET, mNC-FET and AC-FET, and SC-FET and AC-FET after the PSM, respectively.

**Results:** LBR was significant lower in the AC-FET group than that in the mNC-FET (40.0 vs. 43.3%) and SC-FET groups (40.9 vs. 46.5%). The adjusted odds ratios (95% CIs) were 1.11 (1.00, 1.24) for mNC/AC (*P* = 0.044) and 0.84 (0.76, 0.92) for AC/SC (*P* < 0.001), which indicated that the AC-FET group was associated with lowest LBR. The LBR was comparable between matched mNC-FET and SC-FET after adjusting for endometrial thickness. Moreover, a lower clinical pregnancy rate and a higher risk of early pregnancy loss were discovered in AC-FET cycles compared with those in SC-FET.

**Conclusion:** In view of our data, AC used for scheduling FET was associated with lower LBR compared with SC and modified natural cycle. This interpretation requires future verification from well-designed prospective multicenter randomized clinical trials, although the comparisons in our study were conducted in the homogenous population after the PSM.

## Introduction

With the development of cryopreservation technology, the threshold for freezing is falling, and increasing numbers of embryos are being electively frozen and reserved for deferred transfer (1). Compared with fresh embryo transfer, frozen embryo transfer (FET) can provide a more physiologic uterine environment for embryo implantation with a fresh start and regrowth under alternative less intensive endometrial preparation regimens (2). The three most common options, ranging from modified natural cycle (mNC) FET [ovulation triggered by human chorionic gonadotrophin (hCG)], to stimulated cycle (SC) FET, or artificial cycle (AC) FET, have proven to be effective to prepare the endometrium for implantation.

Nowadays, the choice of FET regimens is mainly guided by personal convenience. According to the meta-analysis (3), the endometrium preparation protocols for FET cycles seemed to be equally efficient in terms of clinical pregnancy rate (CPR) and live birth rate (LBR), although the quality of the evidence was low or very low. Quite often, the allocations were not randomized, or the sample size was relatively small, which may lead to potential bias. The first large randomized controlled trial on this topic, including 1,032 patients, was conducted by Groenewoud et al. (4). This non-inferiority trial of mNC-FET vs. AC-FET was concured with previous studies; however, the LBR was 11.5%, which may underestimate the overall situation. In addition, clinicians prefer the predictability, and reliability of AC-FET and have extended its usage to general *in vitro* fertilization population. More recent primary studies are beginning to challenge the comparability of AC-FET and suggest a reduced CPR and LBR compared with those in mNC-FET and SC-FET (5–9).

Hence, in the present study, we conducted this retrospective study aiming to assess the pregnancy outcomes within mNC-FET and AC-FET and SC-FET in a larger cohort. We chose to apply the propensity score matching (PSM) method to implement *post-hoc* randomization. The PSM method is a useful tool to account for imbalance in covariates across groups in observational studies. A propensity score is a single score that represents the probability of receiving a treatment, conditional on a set of observed covariates (10). Individuals with similar propensity scores are then compared across groups.

## Materials and Methods

### Ethical Approval

All procedures performed in studies involving human participants were in accordance with the ethical standards of the institutional and/or national research committee and with the 1964 Helsinki declaration and its later amendments or comparable ethical standards. All participants provided written informed consent.

### Study Design and Sample Selection

The study protocol was approved by the Ethics Committee (Institutional Review Board) of the Shanghai Ninth People's Hospital. We retrospectively analyzed all women who underwent their first autologous frozen–thawed embryo transfer cycles between July 2014 and July 2017 at the Department of Assisted Reproduction of Shanghai Ninth People's Hospital affiliated with Shanghai Jiao Tong University School of Medicine.

**Figure 1:**
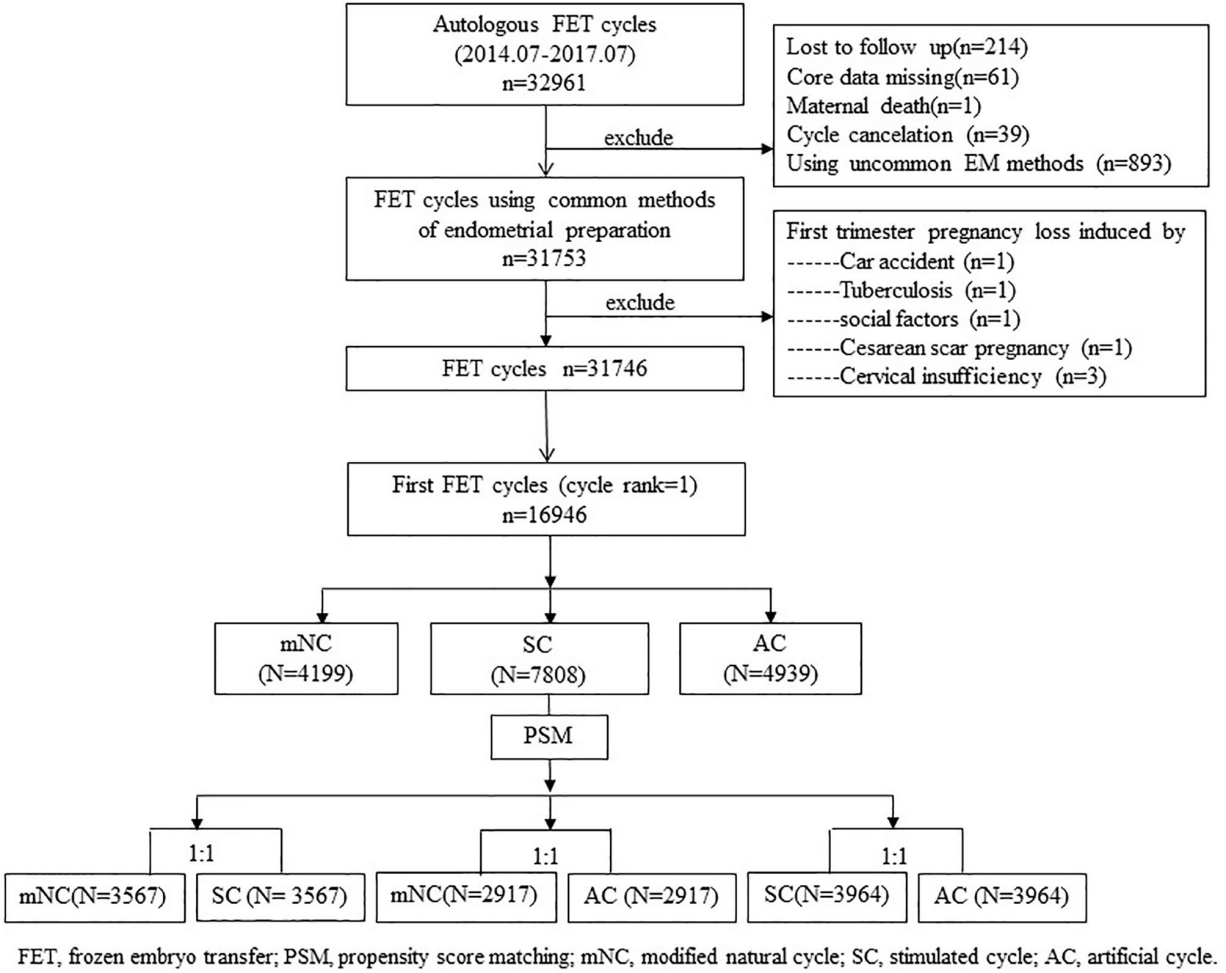
Patient inclusion flowchart.

Database for this study contained cycle-specific information, including endometrial preparation protocols and pregnancy outcomes. No preimplantation genetic screening or diagnosis was performed in our center. The exclusion criteria were cycles experiencing uncommon endometrium preparation protocols, such as true natural cycle or natural cycles receiving vaginal E2, sildenafil, low-dose aspirin to improve endometrial development, etc.; core data missing, such as unknown endometrium preparation protocols; cycle cancellation, and so on. We also excluded cases of induced first trimester pregnancy loss, maternal death, and lost to follow-up. A flow diagram of the patient-selection process is presented in a flowchart (Figure 1).

Observational studies have inherent potential confounders due to lack of randomization (11); therefore, subjects with certain characteristics are more like to be assigned into a treatment group or a comparison group. In our study, PSM using the nearest neighbor matching was established to adjust the baseline features within the three protocols, including maternal age, primary infertility (yes or no), previous pregnancy in the fresh cycle (yes or no), maternal body mass index (BMI), infertility diagnosis, the number of embryos transferred, embryo stage at transfer (cleavage stage or blastocyst), and the embryo quality (good-quality embryo transfer: yes or no) in proportion of 1:1 (mNC-FET vs. SC-FET, mNC-FET vs. AC-FET, SC-FET vs. AC-FET, respectively).

### Observational Indicators and Main Cycle Outcomes

Demographic and reproduction-related clinical parameters of patients in all groups were observed and compared, including maternal age at ovulation retrieval, maternal BMI, primary infertility (yes or no), previous pregnancy in the fresh cycle (yes or no), and infertility diagnosis, which might have biased the results. Furthermore, information on the number of embryos transferred, embryo stage at transfer (cleavage stage or blastocyst), and the embryo quality (good-quality embryo transfer: yes or no) were also available, which might also have affected the results regarding pregnancy outcomes. The cycle characteristics of FET, including endometrial thickness (EMT), were displayed as well and listed as confounding factor in the multivariate regression model. EMT was measured by transvaginal ultrasound scan on the day of hCG administration in mNC-FET cycles or SC-FET cycles. In AC-FET cycles, EMT was recorded from the last ultrasound scan before starting P administration. The maximum distance between two outer edges in the endometrial image of a longitudinal section of the uterus observed by vaginal ultrasound was used to measure (12).

The primary outcome was LBR. A live birth was defined as gestation with breaths or shows any other evidence of life, e.g., heartbeat, umbilical cord pulsation, or definitive movement of voluntary muscle (13).

CPR and abnormal implantation occurrence were our secondary outcomes. A clinical pregnancy (CP) was defined as a pregnancy diagnosed by ultrasonographic visualization of one or more gestational sacs or definitive clinical signs of pregnancy (13). Abnormal implantation was defined as a composite outcome to include biochemical pregnancy, ectopic/heterotopic pregnancy, and first-trimester pregnancy loss (14). A biochemical pregnancy (BP) was defined as a pregnancy diagnosed only by the detection of hCG in serum or urine and that does not develop into a CP (13); an ectopic/heterotopic pregnancy (EP) was defined as the presence of an extra uterine gestation documented by ultrasound or salpingectomy with or without a synchronous intrauterine pregnancy (15); a first-trimester pregnancy loss(early pregnancy loss, EPL) was defined as loss of the entire gestation before 14 weeks of gestation (16).

### Endometrial Preparation Procedures

Endometrial preparation protocols for FET cycles were performed in mNC, SC, or AC. The assignment was not randomized, but was based on physicians' habitual practice and/or patients' preference. It was standard practice in our center and had been described similarly as published recently by our group (17) and are reemphasized as follows.

In the first group, FET was done in a modified natural cycle. We monitored follicular growth by means of serum hormones and transvaginal ultrasound from cycle day 10 onward. Patients were regularly monitored every 2 days. When the diameter of the dominant follicle reached 14 mm, patients were required to perform a urinary LH test at home on the interval day of hospital monitoring. If the urinary LH test was positive, further monitoring of serum hormones and transvaginal ultrasound at hospital were required the same day. When the diameter of the dominant follicle was ≥17 mm and EMT ≥8 mm, with E2 >150 pg/ml and *P* < 1.0 ng/ml, the timing of the embryo transfer was established based on the day of embryo freezing, and the LH value. If LH was <20 IU/l, 5,000 IU hCG was administrated at night (9:00 p.m.) to trigger ovulation and the thaw and transfer of embryos vitrified on day 3 was arranged for 5 days later. If the LH value was ≥20 IU/l, when a spontaneous LH surge was occurring, 5,000 IU hCG was injected the same afternoon when the dominant follicle was scanned and the thaw and transfer was scheduled 4 days later. Similarly, the thaw and transfer of blastocysts was scheduled on the 6th or 7th day according to the same criteria based on serum hormones and ultrasound results. Exogenous progesterone (400 mg/day; Utrogestan; Besins Healthcare, Belgium) was given vaginally starting 2 days after hCG administration.

In the second group, the stimulated method used was as follows: 5 mg letrozole was orally administered daily from cycle days 3–7 to stimulate monofollicular growth, and follicle growth was monitored from cycle day 10. If the diameter of the dominant follicle was <14 mm, the patient received additional HMG 75 units every day. If the diameter of the dominant follicle was ≥14 mm, administration of 5,000 IU hCG and the timing of FET were performed according to the same criteria described above. Specifically, when the dominant follicle reached a mean diameter of ≥17 mm and endometrial thick-ness reached ≥8 mm, with E2 levels preferably >150 pg/ml and progesterone <1 ng/mL, the timing of hCG triggering was dependent on the occurrence of an LH surge. If a serum LHs urge was detected (LH ≥20 IU/L and more than double the average LH level over the past 2 days), 5,000 IU hCG was injected the same afternoon when the dominant follicle was scanned and the day-3 ET was scheduled 4 days later (6 days later for blastocyst transfer). Exogenous progesterone (400 mg/day; Utrogestan; Besins Healthcare, Belgium) was given vaginally starting 2 days after hCG administration. In the absence of an LH surge (LH <20 IU/L), hCG was injected at 9:00 p.m. and ET was arranged 5 days later for 3-day-old embryos or 7 days later for blastocysts. Progesterone exposure was initiated 3 days after ovulatory trigger.

In the third group of AC-FET cycles, oral 17β-estradiol (Fematon 2 mg, three times daily; Abbott Healthcare Products B.V.) was commenced on the second or third day of a natural or P-induced menstrual cycle. Fourteen days later, ultrasound examination was carried out to measure EMT as well as to ensure no dominant follicle emerged. When the EMT attained ≥8 mm, progesterone vaginal suppositories (400 mg/day; Utrogestan; Besins Healthcare, Brussels, Belgium), and oral Fematon yellow tablets (consisted of 2 mg 17β-estradiol and 10 mg dydrogesterone per tablet, 6 mg/day) was initiated. The embryo transfer was performed 3 days after progesterone administration for day-3 embryos or 5 days later for blastocysts. Once a pregnancy was achieved, luteal support was continued to 10 weeks of gestation.

### Statistical Analysis

All data were analyzed using SPSS software version 22.0 (SPSS Inc., Chicago, USA) and R software (R for Windows version 3.6.1). For baseline characteristics and pregnancy outcomes according to FET protocols, hypothesis testing was conducted using Chi-square statistics, Mann–Whitney *U*-tests or Student *t*-tests depending on the research question to be addressed, type and distribution of data and sample size. A *P* < 0.05 was considered statistically significant.

Crude and adjusted odds ratios (ORs) and 95% confidence intervals (CIs) were both displayed. Crude OR was calculated using univariate logistic regression. A multiple logistic regression model was applied to calculate the adjusted odds ratio (aOR) within groups after controlled for EMT.

## Results

A total of 16,946 cycles undergoing *in vitro* fertilization or ICSI who had cryopreserved embryos were evaluated. Of these, there were 4,199, 7,808, and 4,939 mNC-FET, SC-FET, and AC-FET cycles, respectively (Table 1). Furthermore, there were 3,567, 2,917, and 3,964 cycles compared between matched mNC-FET and SC-FET, mNC-FET and AC-FET, and SC-FET and AC-FET after the PSM, respectively. The baseline characteristics were significantly different within groups before the PSM (Table 1). As expected, no significant difference was found between any two of the three groups regarding the baseline characteristics after the PSM. EMT among matched groups were also presented in Table 2. EMT in AC-FET group was demonstrated significantly thinner than mNC-FET and SC-FET group (10.10 ± 2.07 vs. 10.53 ± 2.14 mm, *P* < 0.001; 10.00 ± 2.00 vs. 10.63 ± 2.27 mm, *P* < 0.001, respectively).

**Table 1:** Baseline characteristics of women undergoing frozen embryo transfer (FET) by the different protocols before PSM.

	**SC-FET**	**mNC-FET**	**AC-FET**	**SC vs. NC *P*-value**	**AC vs. NC *P*-value**	**SC vs. AC *P*-value**
*N* (cycle)	7,808	4,199	4,939			
Age (maternal)	31.78 ± 4.51	32.93 ± 4.73	33.6 ± 5.41	<0.001^*^	<0.001^*^	<0.001^*^
BMI (maternal)	21.95 ± 3.19	21.41 ± 2.89	22.06 ± 3.26	<0.001^*^	<0.001^*^	0.128
Primary infertility (YES%)	56.2%	52.3%	44.1%	<0.001^*^	<0.001^*^	<0.001^*^
Previous fresh ET pregnancy (YES%)	0.3%	0.7%	0.1%	0.001^*^	<0.001^*^	0.106
**Cause of infertility (YES%)**						
Male factor	14.0%	12.6%	10.5%	0.027^*^	0.002^*^	<0.001^*^
Ovulatory dysfunction	13.1%	1.8%	13.7%	<0.001^*^	<0.001^*^	0.287
Diminished ovarian reserve	5.4%	7.0%	10.6%	0.001^*^	<0.001^*^	<0.001^*^
Endometriosis	9.6%	12.8%	12.1%	<0.001^*^	0.281	<0.001^*^
Uterine factor	13.2%	13.5%	15.4%	0.618	0.011^*^	<0.001^*^
Tubal factor	69.4%	74.9%	69.2%	<0.001^*^	<0.001^*^	0.843
Other factor	0.2%	0.4%	0.5%	0.058	0.687	0.014^*^
Unknown factor	2.0%	1.5%	1.7%	0.071	0.542	0.221
No. embryos transferred	1.88 ± 0.32	1.87 ± 0.34	1.85 ± 0.36	<0.001^*^	0.011^*^	<0.001^*^
Good-quality embryo (YES%)	95.8%	94.3%	95.2%	<0.001^*^	0.040^*^	0.136
**Embryo stage at transfer**						
Cleavage stage	92.8%	92.1%	93.7%	0.197	0.004^*^	0.047^*^
Blastocyst	7.2%	7.9%	6.3%			

*PSM, propensity score matching; mNC-FET, modified natural cycle; SC-FET, stimulated cycle; AC-FET, artificial cycle*.

* *Statistically significant, with P < 0.05*.

**Table 2:** Baseline characteristics by matched protocols after PSM.

	**SC-FET**	**mNC-FET**	**P^**a**^**	**AC-FET**	**mNC-FET**	**P^**a**^**	**SC-FET**	**AC-FET**	**P^**a**^**
*N* (cycle)	3,567	3,567		2,917	2,917		3,964	3,964	
Age (maternal)	32.33 ± 4.58	32.47 ± 4.48	0.071	33.12 ± 5	33.04 ± 4.74	0.851	32.41 ± 4.53	32.57 ± 4.8	0.447
BMI (maternal)	21.43 ± 2.76	21.44 ± 2.71	0.910	21.57 ± 2.87	21.58 ± 2.85	0.757	21.96 ± 3.16	21.96 ± 3.18	0.752
Primary infertility (YES%)	52.8%	52.8%	0.943	47.1%	47.2%	0.958	47.6%	47.8%	0.910
Previous fresh ET pregnancy (YES%)	0.2%	0.0%	0.059	0.1%	0.1%	1.000	0.2%	0.1%	0.365
**Cause of infertility (YES%)**									
Male factor	13.4%	13.3%	0.835	11.7%	11.6%	0.870	10.3%	10.4%	0.854
Ovulatory dysfunction	1.7%	1.7%	0.928	2.1%	2.0%	0.926	14.5%	13.5%	0.219
Diminished ovarian reserve	6.5%	6.4%	0.923	6.7%	7.1%	0.569	6.1%	6.8%	0.201
Endometriosis	11.2%	11.1%	0.851	12.8%	12.6%	0.875	10.7%	10.9%	0.745
Uterine factor	13.4%	14.0%	0.409	14.0%	13.7%	0.762	13.8%	13.8%	>0.999
Tubal factor	74.0%	74.1%	0.935	75.6%	76.0%	0.737	71.4%	71.9%	0.654
Other factor	0.3%	0.4%	0.413	0.4%	0.5%	0.432	0.3%	0.2%	0.818
Unknown factor	1.4%	1.5%	0.921	1.5%	1.6%	0.749	1.7%	1.6%	0.930
No. embryos transferred	1.87 ± 0.33	1.88 ± 0.33	0.495	1.87 ± 0.33	1.86 ± 0.34	0.188	1.88 ± 0.32	1.89 ± 0.31	0.302
Good-quality embryo (YES%)	95.3%	95.2%	0.867	94.8%	94.8%	0.953	95.9%	96.0%	0.775
**Embryo stage at transfer**									
Cleavage stage	92.3%	92.4%	0.859	93.2%	92.7%	0.473	94.2%	94.3%	0.735
Blastocyst	7.7%	7.6%		6.8%	7.3%		5.8%	5.7%	
Endometrial thickness (EMT, mm)	10.73 ± 2.29	10.54 ± 2.13	<0.001^*^	10.10 ± 2.07	10.53 ± 2.14	<0.001^*^	10.63 ± 2.27	10.00 ± 2.00	<0.001^*^

*PSM, propensity score matching; mNC-FET, modified natural cycle; SC-FET, stimulated cycle; AC-FET, artificial cycle*.

* *Statistically significant, with P < 0.05*.

a *Comparison groups were established by propensity score matching using nearest neighbor matching to adjust the baseline features within the three protocols including maternal age at ovulation retrieval, primary infertility (yes or no), previous pregnancy in the fresh cycle(yes or no), maternal body mass index (BMI), infertility diagnosis, the number of embryos transferred, embryo stage at transfer (cleavage stage or blastocyst), and the embryo quality (good-quality embryo transfer: yes or no)*.

Pregnancy outcomes stratified by matched FET method are shown in Table 3. LBR was significant lower in the AC-FET group than in the mNC-FET (40.0 vs. 43.3%) and SC-FET groups (40.9 vs. 46.5%). The adjusted ORs (95% CIs) were 1.11 (1.00, 1.24) for mNC/AC (*P* = 0.044) and 0.84 (0.76, 0.92) for AC/SC (*P* < 0.001), which indicated that the AC-FET group was associated with lowest LBR. The LBR was comparable between matched mNC-FET and SC-FET after adjusting for EMT.

**Table 3:** Pregnancy outcomes stratified by matched protocols after the PSM.

	**SC-FET**	**mNC-FET**	***P***	**AC-FET**	**mNC-FET**	***P***	**SC-FET**	**AC-FET**	***P***
*N* (cycle)	3,567	3,567		2,917	2,917		3,964	3,964	
Live birth	1,705 (47.8%)	1,618 (45.4%)	0.039^*^	1,167 (40.0%)	1,262 (43.3%)	0.012^*^	1,842 (46.5%)	1,623 (40.9%)	<0.001^*^
Crude OR (95%CI)		0.91 (0.83, 1.00)			1.14 (1.03, 1.27)			0.80 (0.73, 0.87)	
aOR		0.92 (0.84, 1.01)	0.081		1.11 (1.00, 1.24)	0.044^*^		0.84 (0.76, 0.92)	<0.001^*^
Clinical pregnancy	1,990 (55.8%)	1,882 (52.8%)	0.010^*^	1,448 (49.6%)	1,489 (51%)	0.283	2,169 (54.7%)	2,008 (50.7%)	<0.001^*^
Crude OR (95%CI)		0.89 (0.81, 0.97)			1.06 (0.95, 1.17)			0.85 (0.78, 0.93)	
aOR		0.90 (0.82, 0.98)	0.022^*^		1.04 (0.94, 1.16)	0.441		0.88 (0.81, 0.96)	0.006^*^
Abnormal implantation	386 (10.8%)	370 (10.4%)	0.538	361 (12.4%)	314 (10.8%)	0.054	439 (11.1%)	500 (12.6%)	0.034^*^
Crude OR (95%CI)		0.95 (0.82, 1.11)			0.85 (0.73, 1.00)			1.16 (1.01, 1.33)	
aOR		0.95 (0.82, 1.11)	0.533		0.87 (0.74, 1.03)	0.100		1.14 (0.99, 1.31)	0.070
Biochemical pregnancy	134 (3.8%)	132 (3.7%)	0.901	128 (4.4%)	111 (3.8%)	0.261	179 (4.5%)	193 (4.9%)	0.457
Crude OR (95%CI)		0.98 (0.77, 1.26)			0.86 (0.66, 1.12)			1.08 (0.88, 1.33)	
aOR		0.99 (0.78, 1.27)	0.956		0.86 (0.66, 1.12)	0.272		1.08 (0.87, 1.33)	0.499
Ectopic/heterotopic pregnancy	58 (1.6%)	39 (1.1%)	0.052	51 (1.7%)	32 (1.1%)	0.036^*^	64 (1.6%)	66 (1.7%)	0.860
Crude OR (95%CI)		0.67 (0.44, 1.01)			0.62 (0.4, 0.97)			1.03 (0.73, 1.46)	
aOR		0.66 (0.44, 1.00)	0.050		0.65 (0.41, 1.01)	0.057		0.97 (0.68, 1.38)	0.877
First-trimester pregnancy loss	194 (5.4%)	199 (5.6%)	0.795	182 (6.2%)	171 (5.9%)	0.546	196 (4.9%)	241 (6.1%)	0.027^*^
Crude OR (95%CI)		1.03 (0.84, 1.26)			0.94 (0.75, 1.16)			1.24 (1.03, 1.51)	
aOR		1.02 (0.83, 1.25)	0.834		0.96 (0.77, 1.2)	0.736		1.23 (1.01, 1.49)	0.042^*^

*PSM, propensity score matching; mNC-FET, modified natural cycle; SC-FET, stimulated cycle; AC-FET, artificial cycle*.

*The aORs were calculated in the multiple logistic regression model and the covariate was endometrial thickness (EMT) only*.

* *Statistically significant, with P < 0.05*.

As for secondary outcomes, CPR was significantly lower in the AC-FET group than in the SC-FET (50.7 vs. 54.7%). It was also significantly lower in the mNC-FET group compared with SC-FET (52.8 vs. 55.8%). No significant difference was found between matched mNC-FET and AC-FET groups.

In the univariate model, AC-FET group was associated with higher risk of abnormal implantation compared with SC-FET group (crude OR: 1.16 for AC/SC; 95% CI, 1.01–1.33, *P* = 0.034) and slightly but non-significantly higher risk compared with mNC-FET (crude OR: 0.85 for NC/AC; 95% CI, 0.73–1.00, *P* = 0.054). The differences were both non-significant in the multivariate models. When we compared the independent outcomes of abnormal implantation, however, only the comparison of first-trimester pregnancy loss between AC-FET and SC-FET reached the significance (4.9 vs. 6.1%, *P* = 0.042). Results for other individual outcomes were comparable within the matched groups.

## Discussion

Compared with previous studies, our practice based on *post-hoc* randomization and large sample may provide evidence-based guidence for choosing approporiate endometrium preparation protocols for FET. The inclusion of a large cohort (*n* = 3,567, 2,917, 3,964 for pairwise comparisons, respectively) at a single institution meant that the protocols for assisted reproductive techniques were relatively homogeneous (8). Our findings confirmed the hypothesis that the AC was associated with lower LBR than mNC and SC while preparing endometrium for the transfer of frozen-thawed embryos.

Our data added to the current body of evidence and was in line with the recent literatures which found adverse effect of artificial protocol used for scheduling FET on LBR (viable in Table S1) (6–9). AC-FET has attracted attention as an effective protocol preparing endometrium for patients with regular or irregular menses cycles since AC-FET can reduce the need for repeated hospital visits and thus increase patient convenience (2, 6, 18). We found that lower rates of live birth in AC-FET cycles compared to mNC-FET or SC-FET cycles were not affected after adjusting for EMT, though AC-FET was related to a thinner endometrium. Besides, the role of the relatively newer oral ovulation induction agents in SC-FET cycles, aromatase inhibitors in our study, was proved to generate better pregnancy outcomes which was also in line with our results (9, 17).

Possible explanations propounded by previous clinical studies concerned about adverse effects of the inherent “artificial” procedure of AC-FET compared with endogenous steroid hormones in mNC-FET or SC-FET (19). A series of preliminary study have provided new insights about the impact of absence of a corpora lutea (CL) (20–22). One of the studies prospectively compared risk of preeclampsia between groups with different corpus luteum numbers at conceptions and reported that the absence of corpus luteum in an AC resulted in an increased risk of preeclampsia as well as poorer vascular health in early pregnancy compared to modified natural cycle (CL = 1) (20). Likewise, another article from Japan indicated the AC-FET may cause changes in the structure and/or function of the extracellular matrix in the decidual layer and was associated with underdevelopment decidual layer after pregnancies (23). These recent evidences suggest a potential importance of corpus luteum (CL) and it may at least partly be responsible for adverse effect of AC-FET on delivery.

Regarding the CPR and outcomes of abnormal implantation, a lower CPR and a higher risk of early pregnancy loss were discovered in AC-FET cycles compared to SC-FET. The results were also in accordance with previous retrospective study (8). On the one hand, letrozole use in SC-FET cycles reduces serum and intraovarian estrogens by blocking the synthesis of estrogen from androgens in the ovarian granulosa cells leading to optimal endometrial development, therefore better endometrium receptivity and improved CP (9). On the other hand, EMT may be an important mediator, as effects of AC-FET on composite abnormal implantation become non-significant in the multiple logistic regression model after controlling for EMT. A thin endometrial lining has been shown to be associated with lower success rates due to adverse effects on receptivity or endometrial advancement (24).

There are potential weaknesses. The retrospective design could not eliminate the impact of potential risk factors not included. Furthermore, the pregnancy outcomes can be affected by factors in multiple stages. For example, results showed that transfer of a top-quality embryo and the embryo stage may be the most important factors as regards improving the chance of live birth after FET (25, 26). By using the propensity scores matching, we balanced measured covariates that potentially affect LBR across groups, such as maternal age, primary infertility (yes or no), BMI, previous pregnancy in the fresh cycle (yes or no), embryo stage, good-quality embryo transfer (yes or no), and number of embryos transferred. The patients who were auto-matched by this method in this study had the similar basic characteristics, which let the analysis focus on the cycle regimens for FET cycles and reduce other confounders. We chose not to adjust for multiple comparisons so that no meaningful results were missed. Our study's findings were also strengthened by the large cohort, the accuracy of patient and cycle information, and the data processing. Because the laboratory procedures were the same in both groups and the potential bias were avoided to the maximum extent, the results of our study may be considered to be reliable.

## Conclusion

In view of our data, AC used for scheduling FET was associated with lower LBR compared with stimulated cycle and modified natural cycle. It was of value to identify the endometrium preparation protocol as a target role affecting later delivery and was critical for patients and clinicians in making an informed decision on the use of methods for endometrium preparation for a FET. This interpretation requires future verification from well-designed prospective multicenter randomized controlled trials, although the comparing in our study was conducted in the homogenous population after the propensity score matching.

## Data Availability Statement

All datasets generated for this study are included in the article/Supplementary Material.

## Ethics Statement

The studies involving human participants were reviewed and approved by Ethics Committee (Institutional Review Board) of the Shanghai Ninth People's Hospital. The patients/participants provided their written informed consent to participate in this study.

## Author Contributions

YW supervised the entire study, including the procedures, conception, design, and completion. BW and QZ were responsible for the collection of data. BW contributed the data analysis and drafted the article. YW participated in the interpretation of the study data and in the revisions of the article. All authors contributed to the article and approved the submitted version.

## Conflict of Interest

The authors declare that the research was conducted in the absence of any commercial or financial relationships that could be construed as a potential conflict of interest.
