# Supplementary material for: Pregnancy Outcomes After Different Cycle Regimens for Frozen-Thawed Embryo Transfer: A Retrospective Study Using Propensity Score Matching
Source: Front Med (Lausanne). 2020 Jul 28;7:327. doi: 10.3389/fmed.2020.00327 (PMC7399073; doi:10.3389/fmed.2020.00327)
Supplement: Supplementary file 1 [file Table_1.doc]

**Table S1. Summary of selected previous studies on comparisons between endometrial preparation protocols for FET since 2009.**

| **study and year** | **design** | **protocol** | **cycles, n** | **LBR, %** | **CPR, %** | **BPR, %** | **EPR, %** | **EPLR, %** | **main outcome** | **aOR** | **P** |
| --- | --- | --- | --- | --- | --- | --- | --- | --- | --- | --- | --- |
| Saito, K, et al., 2019  Hum Reprod | Retro | AC | 24225 | 21.5% | 32.1% | NA | 0.1% | NA | Obstetrical complications | Increased risks of HDP and placenta accrete; decreased risk of GDM | / |
|  |  | NC | 10755 | 26.0% | 36.1% | NA | 0.2% | NA |  | Ref | Ref |
| Wang, Ange, et al., 2019 Arch Gynecol Obstet | Retro; For euploid embryo | NC | 214 | NA | NA | NA | NA | NA | Ongoing pregnancy rate | 2.05 (1.27–3.31) | 0.003 |
|  |  | AC | 175 | NA | NA | NA | NA | NA |  | Ref | Ref |
| Zhang, et al., 2019  Fertil Steril | Retro;  For PCOS | SC(LE) | 1571 | 54.4% | 60.3% | 6.0% | 6.6% | NA | LBR | 1.33(1.09–1.61) | NA |
|  |  | AC | 1093 | 50.7% | 61.6% | 7.5% | 11.8% | NA |  | Ref | Ref |
| Huang, et al., 2018  Gynecol Endocrinol | Retro | NC | 1838 | 37.5 | 47.7 | NA | NA | NA | CPR and LBR | NA | <.05 |
|  |  | AC | 1666 | 31.5 | 44.5 | NA | NA | NA |  | NA | <.05 |
|  |  | SC(LE) | 340 | 31.8 | 45.6 | NA | NA | NA |  | NA | <.05 |
|  |  | SC(HMG) | 1226 | 41.1 | 54.9 | NA | NA | NA |  | NA | Ref |
| Hatoum, et al., 2018  J Assist Reprod Genet | Retro | SC | 1061 | 59.9 | NA | 13.55 | NA | 16.1 | LBR and EPLR | NA | 0.0001 |
|  |  | AC | 865 | 29.6 | NA | 17.7 | NA | 35.5 |  | NA | Ref |
| Agha-Hosseini, et al., 2018  Turk J Obstet Gynecol | RCT | NC+HCG | 85 | 35.3 | 38.9 | 9.3 | NA | NA | CPR | NA | NS |
|  |  | AC | 85 | 31.8 | 35.3 | 9.6 | NA | NA |  | NA | Ref |
| Tatsumi, et al., 2017  Hum Reprod | Retro | SC(LE) | 2409 | 51.3 | 61.3 | NA | NA | NA | CPR | 1.62 (1.54,1.70) | <0.001 |
|  |  | NC | 41470 | 26.4 | 36.4 | NA | NA | NA |  | NA | NA |
|  |  | AC | 66843 | 23.3 | 33.0 | NA | NA | NA |  | Ref | Ref |
| Shiotani, et al., 2017  Reprod Med Biol | RCT | AC with HCG | 86 | NA | 44.2 | NA | *NA* | NA | Pregnancy and implantation rate | NA | NS |
|  |  | AC without HCG | 87 | NA | 46.0 | NA | NA | NA |  | NA | Ref |
| Aleyasin, et al., 2017  Int J Reprod Biomed | RCT | AC | 50 | 30.0 | 26.0 | 36.0 | NA | NA | LBR | NA | 0.405 |
|  |  | SC(LE+HMG) | 50 | 26.0 | 28.0 | 36.0 | NA | NA |  | NA | Ref |
| Montagut, et al., 2016  Hum Reprod | Retro | NC | 501 | NA | 46.9 | NA | NA | NA | CPR | 2.18 (1.64–2.90) | <0.001 |
|  |  | NC+ LPS | 828 | NA | 39.9 | NA | NA | NA |  | 1.67 (1.31–2.12) | <0.001 |
|  |  | NC+HCG+LPS | 1024 | NA | 29.7 | NA | NA | NA |  | Ref | Ref |
| Orvieto, et al., 2016  J Assist Reprod Genet | Retro | NC+P | 133 | NA | 36.8 | 3.0 | NA | NA | CPR, and ongoing pregnancy rates | NA | <0.001 |
|  |  | AC | 167 | NA | 22.8 | 13.2 | NA | NA |  | NA | Ref |
| Guan, et al., 2016  Syst Biol Reprod Med | Retro | SC(LE) | 132 | 31.8 | 40.9 | NA | 14.8 | 3.7 | LBR | 0.703(0.462,1.072) | 0.101 |
|  |  | AC | 794 | 34.1 | 44.2 | NA | 12.8 | 3.4 |  | 0.753(0.586,0.968) | 0.027 |
|  |  | GnRH agonist+ AC | 129 | 30.2 | 44.2 | NA | 14.0 | 3.5 |  | 0.579(0.375,0.892) | 0.013 |
|  |  | NC | 427 | 43.1 | 50.4 | NA | 9.8 | 0.5 |  | Ref | Ref |
| Groenewoud, et al., 2016  Hum Reprod | RCT | NC+HCG | 495 | 11.5 | 19.0 | NA | NA | NA | LBR | 0.8 (0.53,1.25) | 0.300 |
|  |  | AC | 464 | 8.8 | 16.2 | NA | NA | NA |  | Ref | Ref |
| Greco, et al., 2016  J Assist Reprod Genet | RCT | NC+HCG | 109 | 45.8 | 54.1 | 8.2 | NA | NA | CPR | NA | 0.612 |
|  |  | GnRH-agonist+AC | 113 | 41.5 | 50.4 | 11.5 | NA | NA |  | NA | Ref |
| Peeraer, et al., 2015  Hum Reprod | RCT | SC(HMG) | 221 | 19.9 | 23.5 | NA | NA | NA | Implantation rate | 1.3 (0.9,2.0) | 0.145 |
|  |  | NC | 213 | 14.1 | 17.4 | NA | NA | NA |  | Ref | Ref |
| Mounce, et al., 2015  Fertil Steril | RCT | AC | 73 | 34.2 | 35.6 | NA | NA | NA | LBR | NS | 0.51 |
|  |  | NC | 72 | 29.2 | 33.3 | NA | NA | NA |  | Ref | Ref |
| Yu, et al., 2015  Arch Gynecol Obstet | RCT  For PCOS | AC | 273 | 30.0 | 41.0 | 10.6 | 7.1 | NA | Clinical outcomes | NA | NS |
|  |  | SC(HMG) | 262 | 31.7 | 41.6 | 14.9 | 8.3 | NA |  | NA | Ref |
| Li, et al., 2014  Arch Gynecol Obstet | Retro | NC | 517 | 42.2 | 51.3 | NA | 4.9 | NA | Clinical outcomes | NA | NS |
|  |  | AC | 354 | 32.5 | 44.4 | NA | 5.7 | NA |  | NA | P<0.05 |
|  |  | SC(LE) | 359 | 44.6 | 53.2 | NA | 4.2 | NA |  | NA | Ref |
| Levron, et al., 2014  Gynecol Endocrinol | Retro | NC | 798 | NA | 12.9 | 4.04 | NA | NA | Clinical outcomes | NA | P<0.05 |
|  |  | AC | 437 | NA | 8.5 | 4.44 | NA | NA |  | NA | Ref |
| Hu, et al., 2014  Clinical Endocrinology | Retro | SC(LE) | 40 | NA | 65.0 | NA | NA | NA | CPR | NA | 0.010 |
|  |  | AC | 76 | NA | 40.8 | NA | NA | NA |  | NA | 0.532 |
|  |  | SC(HMG) | 32 | NA | 34.4 | NA | NA | NA |  | NA | Ref |
| Zhang, et al., 2013  Syst Biol Reprod Med | Retro | NC | 2198 | NA | 24.8 | NA | 4.9 | NA | EPR | NA | 0.045 |
|  |  | AC | 1836 | NA | 29.7 | NA | 5.8 | NA |  | NA | Ref |
| Xiao, et al., 2012  Syst Biol Reprod Med | Retro | NC | 380 | NA | 37.89 | 5.26 | NA | NA | CPR | 1.12(0.86,1.45) | 0.006 |
|  |  | AC | 646 | NA | 35.29 | 8.36 | NA | NA |  | Ref | Ref |
| Hancke, et al., 2012  J Assist Reprod Genet | Retro | NC+HCG | 148 | 20.9 | 34.5 | NA | NA | NA | CPR | 1.88(0.91,3.89) | 0.150 |
|  |  | AC | 55 | 12.7 | 21.8 | NA | NA | NA |  | Ref | Ref |
| Tomas, et al., 2012  Fertil Steril | Retro | AC | 2,858 | 20.1 | 27.7 | 6.6 | NA | NA | Pregnancy loss | NA | <.0001 |
|  |  | NC+HCG | 444 | 23.5 | 29.1 | 6.4 | NA | NA |  | NA | <.0001 |
|  |  | NC+P | 1,168 | 20.7 | 24.3 | 2.4 | NA | NA |  | NA | Ref |
| Chang, et al., 2011  J Assist Reprod Genet | Retro | AC | 204 | NA | 30.4 | 4.4 | NA | NA | CPR | 0.567(0.379,0.847) | 0.006 |
|  |  | NC+HCG | 134 | NA | 41.8 | 2.2 | NA | NA |  | 0.683(0.435,1.073) | 0.098 |
|  |  | NC | 310 | NA | 41.9 | 2.6 | NA | NA |  | Ref | Ref |
| Hill, et al., 2010  Fertil Steril | Retro | GnRH agonist+AC | 1151 | 33.1 | 49.7 | 58.7 | NA | NA | LBR | 1.42 (1.06,1.90) | <0.01 |
|  |  | NC | 240 | 23.4 | 39.0 | 68.6 | NA | NA |  | Ref | Ref |
| Givens, et al., 2009  Reprod Biomed Online | Retro | NC | 862 | 28.4 | 33.4 | 9.6 | 0.2 | NA | CPR | NA | 0.011 |
|  |  | AC | 262 | 29.4 | 40.5 | 13 | 0.8 | NA |  | NA | Ref |

Note: Retro: retrospective; Ref: reference; NS: no significant differences; CPR: clinical pregnancy rate; BPR: biochemical pregnancy rate; EPR: ectopic pregnancy rate; EPLR: early pregnancy loss; LBR: live birth rate
